# Underwater dam image enhancement based on CNN-transformer fusion

**DOI:** 10.1038/s41598-025-23746-w

**Published:** 2025-11-12

**Authors:** Zhenggang Yang, Chongxin Yuan, Luyao Li, Peng Ding, Junjie Zou, Kunpeng Wang, Hongjin Zhu

**Affiliations:** 1 POWERCHINA GuiYang Engineering Corporation Limited, Guiyang, 550081 China; 2 School of Computer Science, China West Normal University, Nanchong, 637009 China; 3 Present Address: Key Laboratory of Earth Exploration and Information Technology of Ministry of Education, Chengdu University of Technology, Chengdu, 610059 China

**Keywords:** Underwater Image Enhancement, Convolutional Neural Network (CNN), Vision Transformer, Generative Adversarial Network (GAN), Feature Fusion, Energy science and technology, Engineering, Mathematics and computing

## Abstract

In the field of hydropower engineering, the safety inspection of underwater dam structures relies heavily on high-precision image analysis. However, images captured by underwater robots generally suffer from optical degradation issues, such as speckle noise interference, blue-green color shift, low contrast, and blurred details. Traditional image denoising algorithms fail to fully account for the optical transmission characteristics of the underwater environment, often leading to the loss of critical structural detail information when processing dam underwater images. Meanwhile, existing deep learning-based image processing methods, which lack comprehensive modeling of the physical process of underwater image degradation, struggle to achieve ideal results in color restoration and edge preservation. These image quality issues may cause misjudgments in dam defect detection, seriously affecting the accuracy of safety assessments. To address the above problems, this study proposes an innovative image denoising and super-resolution network, namely Enhanced Super-Resolution Transformer GAN (ESRTGAN), which integrates CNN-based local feature extraction and Transformer-based global context modeling to tackle dam underwater image degradation. By effectively fusing the local feature extraction capability of Convolutional Neural Networks (CNN) and the global context modeling capability of Vision Transformer, ESRTGAN achieves high-quality image restoration and enhancement. The network adopts multi-scale feature fusion strategy, adaptive channel attention mechanism, and progressive training method, significantly improving the reconstruction capability of image details. Experimental results show that ESRTGAN exhibits excellent performance in terms of PSNR, SSIM, and LPIPS metrics in the enhancement experiments on real dam underwater image datasets while maintaining high computational efficiency. Additionally, the processed images meet the requirements of manual interpretation in subjective visual effects, providing reliable technical support for the automated analysis of long-term dam health monitoring.

## Introduction

In the field of hydropower engineering, full-lifecycle health monitoring of underwater dam structures holds irreplaceable strategic significance for ensuring stable energy output and public safety in downstream areas. Restricted by the physical constraints of underwater operation spaces and the efficiency bottlenecks of traditional manual inspection methods, computer vision-based detection technology using underwater robots as carriers is reshaping industry standards^1,2^. Underwater robot detection systems not only significantly reduce labor costs but also provide more reliable decision-making support for engineering operation and maintenance by enhancing inspection efficiency and data collection density^3^.

Endowed with advantages such as non-contact operation and strong adaptability to complex environments, underwater inspection robots have become key equipment for acquiring images of underwater dam structures. Notably, the quality of these acquired images directly determines the accuracy of defect identification and safety assessment. Nevertheless, the complex underwater environment poses multiple challenges to image acquisition: First, the optical transmission characteristics of water lead to image quality degradation. Water exhibits strong absorption of red light and weak absorption of blue and green light, resulting in significant blue-green color cast in images along with reduced contrast^4,5^. Second, noise interference is prominent, including inherent electronic noise of cameras, speckle noise caused by suspended particles in water, and motion blur induced by robot movement^6^. Third, extracting structural features is highly challenging. Critical defects on dam surfaces, such as cracks and corrosion, are extremely small in scale and easily obscured by noise. Traditional denoising algorithms, which lack targeted handling of underwater optical properties, tend to blur structural edges while suppressing noise—this in turn undermines the reliability of subsequent defect detection^7^.

In the context of underwater image denoising, conventional methods—such as those based on wavelet transform, partial differential equations (PDEs), and various filtering algorithms—exhibit considerable limitations when addressing complex underwater noise. While wavelet transform can mitigate noise to a certain extent, it tends to cause information loss in texture-rich regions and demonstrates poor adaptability to complex noise models^8^. PDE-based methods suffer from high computational complexity, time-consuming solution processes, and inadequate real-time performance^9^. Mean filtering blurs image details, whereas median filtering is prone to edge jitter when dealing with large-area noise^10^.

In recent years, the rise of deep learning has brought new opportunities for underwater image denoising and enhancement. Deep learning-based image processing methods have demonstrated exceptional performance in numerous computer vision tasks^11–13^. Some researchers have attempted to apply simple Convolutional Neural Network (CNN) architectures to underwater image denoising. By training on datasets composed of numerous pairs of synthetic underwater noisy images and their corresponding clean counterparts, these approaches have achieved effective suppression of common underwater noise^14^. Zhang et al.^15^ constructed a Feed-Forward Denoising Convolutional Neural Network (DnCNN) to handle Gaussian denoising with unknown noise levels. Wang et al.^16^ proposed an end-to-end underwater image enhancement network, UIE-Net, which consists of color correction and defogging sub-networks. This network enables the joint learning of color enhancement and defogging tasks, reducing the impact of fine textures and color noise on feature extraction. Sun et al.^17^ designed an underwater image enhancement network based on an encoder-decoder structure, which yields remarkable results in enhancing turbid underwater images. li et al.^18^ integrated image physical models with underwater optical properties to build UIEBD, a large-scale benchmark dataset for underwater image enhancement containing 950 images. This dataset covers different water types and degradation levels. Based on UIEBD, they trained Water-Net, a gated fusion network that enhances images via white balance, histogram equalization, and gamma correction algorithms. It fuses confidence maps of different results to generate the final image, and leverages CNNs and perceptual loss functions to learn latent attributes of underwater images, thereby improving image fusion performance. Qi et al.^19^ proposed the Underwater Image Collaborative Enhancement Network (UICoE-Net), a model that combines a Siamese structure with an encoder-decoder framework. To strengthen the interdependencies between different branches of the Siamese architecture, they embedded feature matching modules into every layer of the structure, enabling more effective information interaction across branches.

As attention mechanisms have shown remarkable performance in advanced visual tasks, researchers have integrated them into underwater image enhancement algorithms to help the model focus on essential information selectively. One notable example is Semi-UIE, a semi-supervised underwater image enhancement framework. This framework makes use of both unlabeled data and a small amount of labeled data to greatly boost its generalization ability. Built on the UNet architecture, it incorporates a new aggregated attention mechanism that leverages multi-scale convolutional kernels to achieve efficient feature aggregation. This design not only enhances the clarity and realism of underwater images but also maintains high computational efficiency. Additionally, Zamir et al.^20^ introduced a supervised attention module to assist in feature selection, while Chen et al.^21^ proposed Feature Attention, a mechanism that merges channel attention and pixel attention. By treating various features and pixels with different strategies, this approach enhances the model’s adaptability when processing diverse types of data.

Self-attention networks have become increasingly popular in tackling various computer vision tasks, mainly because they can be trained jointly with the main model and deliver excellent performance. KiT et al.^22^ developed a method for non-local interactions by implementing a pairwise local attention mechanism. This mechanism retains the inductive bias of locality while introducing non-local connections, and its computational complexity increases linearly with the spatial resolution of the input image. Chen et al.^23^ created a rectangular window self-attention mechanism for image restoration. This mechanism improves the aggregation of information between different windows and ensures that the model remains computationally efficient. Li et al.^24^ proposed anchored stripe self-attention, which effectively expands the modeling range of self-attention beyond local areas. Zhou et al.^25^ put forward HCLR-Net, which extracts features efficiently and restores texture details through an adaptive hybrid attention module and a dedicated detail restoration branch. Moreover, Xu et al.^26^ designed an underwater image enhancement algorithm based on cross-attention, which can alternately capture both local and global information of underwater images. Zhang et al.^27^ also introduced a two-layer attention mechanism that combines spatial attention and channel attention, aiming to enhance color restoration while preserving the key features of the image.

Compared with methods that rely on manually designed functions for global adjustment of image color and contrast, the aforementioned deep learning approaches utilize their robust nonlinear mapping capabilities to learn higher-level features and extract rich image information. This allows them to more accurately address degradation issues such as underwater scattering and color distortion, achieving refined underwater image enhancement. However, these methods are highly dependent on high-quality underwater image datasets, and the current scarcity of such datasets remains an urgent challenge to be resolved. Additionally, CNN models trained for specific underwater scenarios generally exhibit insufficient generalization capabilities when processing underwater images with different types of degradation.

With in-depth research, a series of innovative deep learning denoising and enhancement methods have emerged. Goodfellow introduced Generative Adversarial Networks (GANs), a pioneering concept that uses adversarial training between a generator and a discriminator to skillfully learn data distributions and generate high-quality images^28^. Subsequently, GANs have been extensively studied in the field of image generation and widely applied in image generation, enhancement/restoration, and style transfer^29,30^.

A typical GAN consists of a generator and a discriminator, which optimize their outputs through adversarial gameplay. Training a GAN requires a dataset containing pairs of underwater images and their corresponding high-quality reference images. During training, the generator strives to generate results highly similar to real images, while the discriminator distinguishes between real and fake images and outputs a probability value between 0 and 1. If the generated results fail to deceive the discriminator, the generator continues learning until it achieves “fake-but-real” performance. This process essentially solves a minimax problem—minimizing the generator’s loss function and maximizing the discriminator’s loss function—ultimately reaching a Nash equilibrium^31^. After training, the generator can be directly used for underwater image enhancement.

In recent years, the success of Transformers has prompted researchers to explore their potential in computer vision (CV). For a long time, CNNs dominated traditional CV tasks by extracting local image features through convolution and pooling operations, but they have limitations in modeling global context information. The emergence of Vision Transformer (ViT)^32^ broke the reliance on CNNs in the CV field and first demonstrated the effectiveness of pure Transformer architectures for image classification tasks. ViT divides an image into multiple patches, treats these patches as tokens in natural language processing (NLP), and converts them into sequences via linear projection for input into a Transformer encoder. ViT achieved performance comparable to or better than traditional CNNs in the ImageNet image classification task, opening up new avenues for the CV field. IPT (Image Processing Transformer)^33^ innovatively applied the Transformer architecture to various image restoration tasks, including denoising, super-resolution, and defogging. IPT adopts a multi-head and multi-tail structure, trains on diverse corrupted image pairs generated from the large-scale ImageNet dataset, and introduces contrastive learning to achieve strong generalization. Restormer^34^ optimized the Transformer for high-resolution image restoration tasks: it proposes a multi-depth convolutional head transposed attention module, which obtains attention maps by calculating the cross-covariance between feature channels, effectively aggregating local and non-local pixel interactions and reducing computational complexity, making it suitable for processing large-size images. These studies demonstrate that Transformers exhibit unique advantages in the field of image restoration—their powerful global context modeling capability can effectively capture long-range dependencies in images, compensating for the limitations of CNNs in local feature extraction and providing a new technical path for improving image restoration quality.

Islam proposed a real-time underwater image enhancement model (FUnIE-GAN) based on Conditional GAN (cGAN), which models underwater image enhancement as a style transfer problem^35^. The model learns the nonlinear mapping between images by iteratively solving a minimax problem between the generator and discriminator and constructs a perceptual loss function (including global similarity, image content, and local texture loss) to supervise adversarial training. Yang used a dual-discriminator based on the cGAN method to capture local and global semantic information, achieving better performance than existing underwater image enhancement methods on both real and synthetic underwater images and improving image visibility and visual effects^36^. Lu proposed a multi-scale network (MCycleGAN) for underwater image restoration, combining dark channel prior with CycleGAN characteristics: it first obtains a transmission map via dark channel prior, then inputs it into a multi-scale network to learn mapping relationships, and designs an adaptive structural similarity loss to achieve color correction and contrast enhancement while preserving edge structure information^37^. Zhang proposed an end-to-end dual generative adversarial network (DuGAN) for underwater image enhancement, dividing training data into clear and blurry parts and performing adversarial training on different image regions using two discriminators with different strategies^38^. The loss function optimizes iterations by combining content loss, adversarial loss, and style loss, but this method requires specific preprocessing to obtain training data, limiting its applicability to new underwater images. Wang used an Enhanced Super-Resolution Generative Adversarial Network (ESRGAN) for image denoising and enhancement, achieving better visual quality and more realistic textures, winning the PIRM2018-SR Challenge and demonstrating excellent performance in improving image super-resolution effects^39^. However, the adaptability of existing GAN methods to complex underwater environments still needs improvement.

ESRGAN^39^ adopts a pure CNN architecture with RRDB blocks, focusing on super-resolution and local texture enhancement. However, its lack of global context modeling leads to incomplete restoration of large-scale structural features in dam underwater images: for example, it fails to connect scattered crack segments into continuous paths on dam spillway surfaces, or to integrate fragmented texture information of dam foundations. Restormer^34^ addresses global modeling via Transformer-based multi-scale attention, which mitigates turbidity-induced blurring. Yet its dynamic window shifting design causes high computational complexity, making it unsuitable for real-time underwater robot detection; additionally, its over-reliance on global attention blurs local defects, prioritizing global consistency over fine-grained details. Our proposed ESRTGAN resolves these issues by fusing LightViT modules with RRDB blocks: the 7$\times$7 window-based attention in LightViT captures long-range dependencies filling ESRGAN’s gap, while the Attentional Feature Fusion (AFF) module dynamically weights features to avoid Restormer’s local blurring and high complexity.

Considering that dam underwater images have complex edge structures, diverse noise types, and rely on local feature restoration, the CNN-based architecture of ESRGAN alone cannot fully capture long-range dependencies between channels and global structural information. Although ViT demonstrates powerful global context modeling capabilities in image classification, it lacks sensitivity to local details in low-level visual tasks. Therefore, this study proposes an innovative network ESRTGAN. First, the network implements a CNN-Transformer fusion architecture with dynamic weight adaptation, addressing the issue of “difficulty in balancing local details and global correlations”. Meanwhile, it innovatively adopts the AFF dynamic weight module, which can adaptively adjust the feature weights of the CNN (RRDB module) and Vision Transformer (LightViT module) according to the degradation degree of underwater images. Second, targeting the physical degradation mechanisms of underwater optics, the network employs different network modules for adaptation. Third, the network reduces computational complexity by using fixed-window attention combined with attention head sparsification, and replacing fully connected layers with global average pooling (GAP). The network improve the reconstruction of image details, providing a new technical path for high-precision detection of dam underwater images.

## Methodology

### Network architecture

This section first describes the architecture of the proposed ESRTGAN, followed by detailed discussions of the generator and discriminator.

**Fig. 1:**
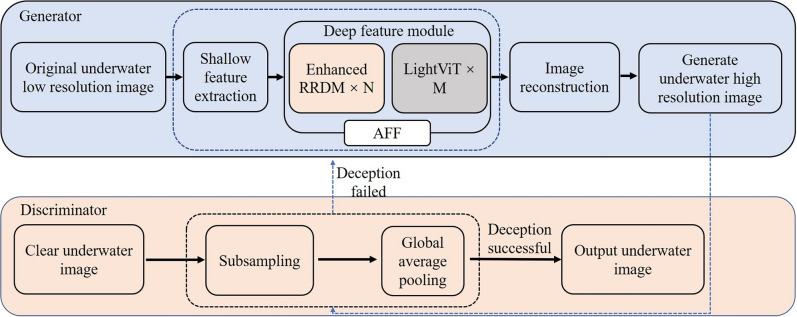
Schematic diagram of the ESRTGAN network structure.

ESRTGAN adopts a GAN framework, consisting of a generator and a discriminator. The generator converts low-resolution, noisy input images into high-resolution, noise-free output images, while the discriminator distinguishes between generated images and real high-resolution images. The generator and discriminator engage in adversarial training: the generator learns to generate more realistic images, and the discriminator continuously improves its discrimination ability, ultimately reaching a Nash equilibrium^31^. The core innovation of ESRTGAN lies in the organic fusion of CNN’s local feature extraction capability and Vision Transformer’s global context modeling capability (Fig. 1).

**Fig. 2:**
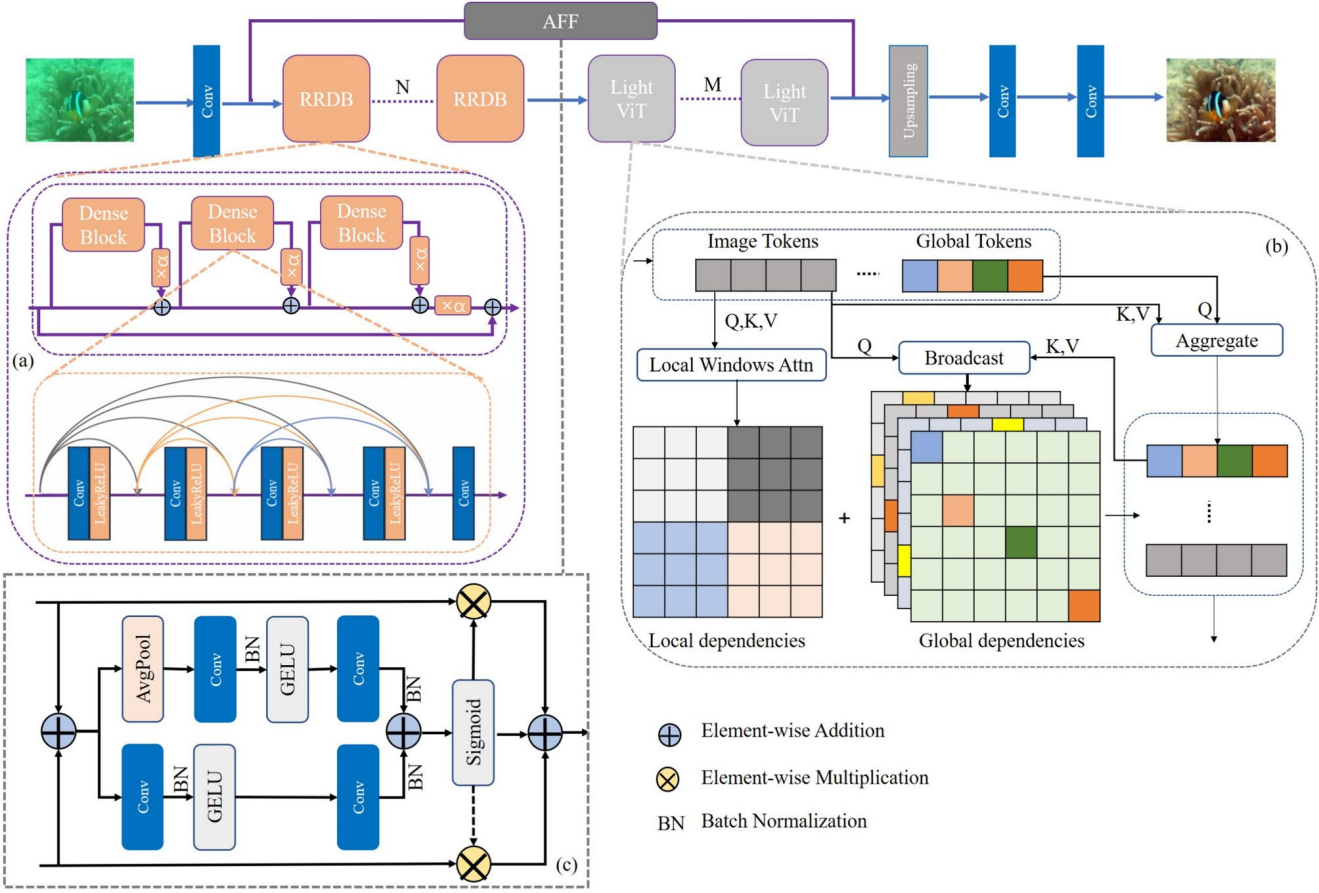
Generator network structure diagram. (**a**) is the implementation process of RRDB^39^, (**b**) is the implementation process of lightvit, (**c**) is Attentional Feature Fusion.

### Generator

The generator comprises three modules: a shallow feature module, a deep feature module, and an image reconstruction module (Fig. 2). The shallow feature module extracts shallow features of the input image using a 3$\times$3 convolutional layer, which can be expressed as:

1 $$\begin{aligned} {F_{shallow}} = {{\mathop {\textrm{Conv}}\nolimits } _{3 \times 3}}({I_{input}}) \end{aligned}$$

where $I_{input} \in \mathbb {R}^{C \times H \times W}$ (C = number of channels, H = height, W = width, $\mathbb {R}$ = real number set), and $\text {Conv}_{3 \times 3}$ denotes the 3$\times$3 convolution operation.

The deep feature module adopts a multi-scale feature fusion strategy, consisting of 8 Enhanced Residual Dense Blocks (RRDB) and 8 lightweight Transformer (LightViT) modules cascaded alternately. Compared to ESRGAN’s deep module (pure RRDB blocks), ESRTGAN’s integration of LightViT modules enables global context modeling: while ESRGAN only perceives local features within 3$\times$3 convolution kernels, LightViT’s 7$\times$7 window attention captures correlations between distant pixel pairs, solving ESRGAN’s structural fragmentation issue. Compared to Restormer’s dynamic window shifting, LightViT uses fixed 7$\times$7 windows to reduce FLOPs, meeting real-time requirements. Additionally, the AFF module dynamically weights CNN/Transformer features: for turbid regions, it increases LightViT weight to enhance global clarity.

Each RRDB contains 3 Dense Blocks^40^ with dense connections (growth rate = 32), combined with residual connections and Batch Normalization (BN) to gradually extract higher-level feature representations (Fig. 2(a)). A single residual block can be expressed as:

2 $$\begin{aligned} {F_{residual}} = {F_{input}} + {\mathop {\textrm{BN}}\nolimits } ({\mathop {\textrm{ReLU}}\nolimits } ({\mathop {\textrm{Conv}}\nolimits } ({F_{input}}))) \end{aligned}$$

where $F_{input}$ is the input feature of the residual block, $\text {BN}$ denotes Batch Normalization, and $\text {ReLU}$ is the activation function. The dense connection within a Dense Block is expressed as:

3 $$\begin{aligned} {F_{dense}} = {\mathop {\textrm{Concat}}\nolimits } ({F_{input}},{\mathop {\textrm{Conv}}\nolimits } ({F_{input}}),{\mathop {\textrm{Conv}}\nolimits } ({\mathop {\textrm{Concat}}\nolimits } ({F_{input}},{\mathop {\textrm{Conv}}\nolimits } ({F_{input}}))), \ldots ) \end{aligned}$$

where $\text {Concat}$ denotes the channel-wise concatenation operation.

The LightViT module uses a fixed 7$\times$7 window partition and 8 attention heads. It calculates attention scores via Query (Q), Key (K), and Value (V) projections to capture global context information of the image (Fig. 2(b)). For the feature map after residual processing ($F_{residual}$), its channel dimension is divided into h heads, followed by Q, K, V projections (using convolutional layers). Q, K, and V are then split by heads to obtain attention scores:

4 $$\begin{aligned} {\mathop {\textrm{Attention}}\nolimits } (Q,K,V) = {\mathop {\textrm{Softmax}}\nolimits } \left( \frac{{Q{K^T}}}{{\sqrt{{d_k}} }}\right) V \end{aligned}$$

where $d_k$ is the dimension of Q/K. The multi-head self-attention output is:

5 $$\begin{aligned} {\mathop {\textrm{MultiHead}}\nolimits } (Q,K,V) = {\mathop {\textrm{Concat}}\nolimits } ({{\mathop {\textrm{Head}}\nolimits } _1},{{\mathop {\textrm{Head}}\nolimits } _2}, \ldots ,{{\mathop {\textrm{Head}}\nolimits } _h}){W^O} \end{aligned}$$

where $\text {Head}_i = \text {Attention}(QW_i^Q, KW_i^K, VW_i^V)$ ($W_i^Q, W_i^K, W_i^V, W^O$ are learnable projection matrices). The output is then reshaped back to the original feature map shape and normalized via Layer Normalization (LN):

6 $$\begin{aligned} F_{LN} = \text {LN}(F_{MultiHead}) = \gamma \cdot \frac{F_{MultiHead} - \mu }{\sqrt{\sigma ^2 + \varepsilon }} + \beta \end{aligned}$$

Where $\mu$ and $\sigma ^2$ are the mean and variance calculated along the channel dimension, $\varepsilon$ is a small constant (to avoid division by zero), and $\gamma$ and $\beta$ are learnable scaling and shifting parameters.

Finally, the feature map is processed via a Multi-Layer Perceptron (MLP):

7 $$\begin{aligned} F_{MLP}= & \text {GELU}(\text {Conv}_{1 \times 1}(F_{LN})) \end{aligned}$$

8 $$\begin{aligned} F_{LightViT}= & \text {Conv}_{1 \times 1}(F_{MLP}) \end{aligned}$$

Thus, the deep feature module can be expressed as:

9 $$\begin{aligned} F_{deep} = \text {AFF}(F_{RRDB}, F_{LightViT}) \end{aligned}$$

where $F_{RRDB}$ is the output of the RRDB module, $F_{LightViT}$ is the output of the LightViT module, and $\text {AFF}$ is an adaptive fusion module. The weights of AFF are dynamically generated from input features via convolutional layers to fuse RRDB local features and LightViT global features (Fig. 2(c)):

10 $$\begin{aligned} F_{AFF}= & \alpha \cdot F_{RRDB} + (1 - \alpha ) \cdot F_{LightViT} \end{aligned}$$

11 $$\begin{aligned} \alpha= & \text {Sigmoid}(\text {Conv}_{1 \times 1}(\text {Concat}(F_{RRDB}, F_{LightViT}))) \end{aligned}$$

where $\alpha$ is the dynamic attention weight, and $\text {Sigmoid}$ is the activation function for normalizing weights to the range [0, 1].

The image reconstruction module reconstructs feature maps into high-resolution images via 2$\times$ upsampling (pixel shuffle) and 3$\times$3 convolution operations:

12 $$\begin{aligned} I_{output} = \text {Conv}_{3 \times 3}(\text {PixelShuffle}(F_{deep}, 2)) \end{aligned}$$

where $\text {PixelShuffle}(F, 2)$ denotes 2$\times$ upsampling of the feature map *F*.

Comprehensively, the generator of ESRTGAN can be expressed as:

13 $$\begin{aligned} I_{output} = \text {Reconstruct}(\text {DeepFeature}(\text {ShallowFeature}(I_{input}))) \end{aligned}$$

### Discriminator

ESRTGAN guides the generator’s optimization through adversarial training: the generator generates high-resolution images, and the discriminator receives both generated images and real high-resolution images to determine whether the input image is real or generated. The discriminator of ESRTGAN is designed as a 5-layer progressive downsampling convolutional network, tailored to the unique characteristics of dam underwater images that generic discriminators often fail to capture. The first four layers are convolutional layers with a fixed kernel size of 3$\times$3 (padding = 1) to match the scale of typical dam defects, ensuring the network can extract fine-grained texture differences between real and fake images. These layers feature progressive channel expansion (from 16 to 128) and a stride of 2—this design achieves downsampling without relying on pooling layers, which would otherwise blur local defect details (a critical limitation for dam safety inspection). Each convolutional layer is followed by LeakyReLU activation with a negative slope of 0.2 and BN with a momentum of 0.9 and epsilon of 1e-5. The fifth layer combines a GAP layer and a single fully connected layer to output the probability that the input image is real. The GAP layer replaces traditional fully connected layers to reduce the discriminator’s parameter, avoiding overfitting to trivial background noise and ensuring the network focuses on critical defect features.

Specifically, the discriminator implements downsampling directly via convolutional layers (avoiding detail loss caused by traditional pooling layers), making it more sensitive to local structural differences in images. It then uses BatchNorm to stabilize training, alleviate gradient vanishing, and enforce feature distribution standardization, improving the discriminator’s generalization to images of different scales.

The discriminator’s feature extraction process can be expressed as:

14 $$\begin{aligned} F_d = \text {LeakyReLU}(\text {BN}(\text {Conv}(I_{input}))) \end{aligned}$$

GAP is then used to reduce the number of parameters:

15 $$\begin{aligned} F_{global} = \frac{1}{H \times W} \sum _{i=1}^H \sum _{j=1}^W F_d(i, j) \end{aligned}$$

where $F_{global}$ denotes the global feature vector, and $H \times W$ is the spatial size of the feature map.Finally, the LeakyReLU activation function and Sigmoid normalization (to [0, 1]) are used to output the probability that the image is a real sample:

16 $$\begin{aligned} P_{real} = \text {Sigmoid}(\text {LeakyReLU}(\text {FC}(F_{global}))) \end{aligned}$$

Through this design, the ESRTGAN discriminator effectively captures multi-scale features of images, making judgments layer by layer from local textures to global structures, and provides strong supervision signals for the generator, ultimately achieving high-quality image super-resolution reconstruction.

### Loss function

The designed loss function integrates adversarial loss and content loss. Adversarial loss forces the generator to generate images (SR) that the discriminator mistakes for real images (HR). The discriminator’s loss function is:

17 $$\begin{aligned} \mathcal {L}_D = -\mathbb {E}_{I_{HR} \sim p_{data}} \log (D(I_{HR})) - \mathbb {E}_{I_{LR} \sim p_{data}} \log (1 - D(G(I_{LR}))) \end{aligned}$$

The generator’s adversarial loss is:

18 $$\begin{aligned} \mathcal {L}_{G_{adv}} = -\mathbb {E}_{I_{LR} \sim p_{data}} \log (D(G(I_{LR}))) \end{aligned}$$

Content loss ensures that the content of the generated image is similar to the real image, calculated using the Euclidean distance between feature maps of generated and real images (extracted via a pre-trained VGG network):

19 $$\begin{aligned} \mathcal {L}_{content} = \frac{1}{C \times H \times W} \Vert \phi (G(I_{LR})) - \phi (I_{HR}) \Vert \end{aligned}$$

where $\phi (\cdot )$ denotes the feature extraction function of the pre-trained VGG network.

The total loss function of the generator is a weighted fusion of adversarial loss and content loss, balancing “visual realism” and “content fidelity” via a weight coefficient $\lambda$:

20 $$\begin{aligned} \mathcal {L}_G = \mathcal {L}_{G_{adv}} + \lambda \cdot \mathcal {L}_{content} \end{aligned}$$

where $\lambda$ is set to 0.05, referring to common parameter settings for GAN models in similar image enhancement tasks^39^, which effectively balances adversarial training stability and content reconstruction accuracy in this study.

### Analysis of network design rationality

The degradation of underwater images essentially stems from the physical process of light transmission in water, manifesting as scattering effects, absorption effects, and superimposed complex noise. The design of ESRTGAN, which fuses CNNs and Vision Transformer, is not a mere technical combination but a “targeted optimization” tailored to the aforementioned physical characteristics.

Scattering effects disrupt the spatial continuity between image pixels, and their physical essence lies in “preserving short-range pixel dependencies while breaking long-range ones.” The 3$\times$3 convolution kernel adopted in the RRDB module has a receptive field that can capture undamaged local texture features in scattered regions; meanwhile, the dense connections in RRDB retain local detail information through channel concatenation, preventing scattered noise from obscuring key defect features. The AFF module generates dynamic weights via Sigmoid activation: in regions with severe scattering, it increases the weight of LightViT global features to enhance the integration of fragmented details; in regions with mild scattering, it raises the weight of RRDB local features to prioritize the preservation of fine textures.

To address absorption effects, a strategy combining channel distribution correction and global color balance is employed. The BN operation after each convolutional layer in RRDB dynamically normalizes the overly strong features of the blue and green channels—specifically, reducing their mean values to around 0, suppressing variance fluctuations, and simultaneously enhancing the weak features of the red channel by adjusting its mean value to match those of the blue and green channels. This essentially compensates for the “channel imbalance caused by absorption” through a data-driven approach. The 8 attention heads in LightViT can capture “inter-channel correlation dependencies” and adjust the channel feature distribution via Layer Normalization, enabling the model to learn the “global law of absorption compensation” and avoiding residual color casts that occur when pure CNNs rely solely on local channel correction.

For complex noise, a dual approach of local denoising and global noise processing is adopted. The residual structure of RRDB separates “useful local features” from “local noise” through element-wise addition of “input features + convolutional outputs.” The MLP layer in LightViT processes and extracts global noise features via the GELU activation function, avoiding the over-smoothing of “non-local noise” that is common in pure CNNs. The 3$\times$3 convolution and GAP layer in the discriminator can accurately distinguish between “real defect details” and “noise-induced pseudo-features”; through adversarial loss, the generator is forced to output results that conform to the “physical distribution of noise-free underwater images,” indirectly enhancing the noise suppression capability of the fused architecture.

**Fig. 3:**
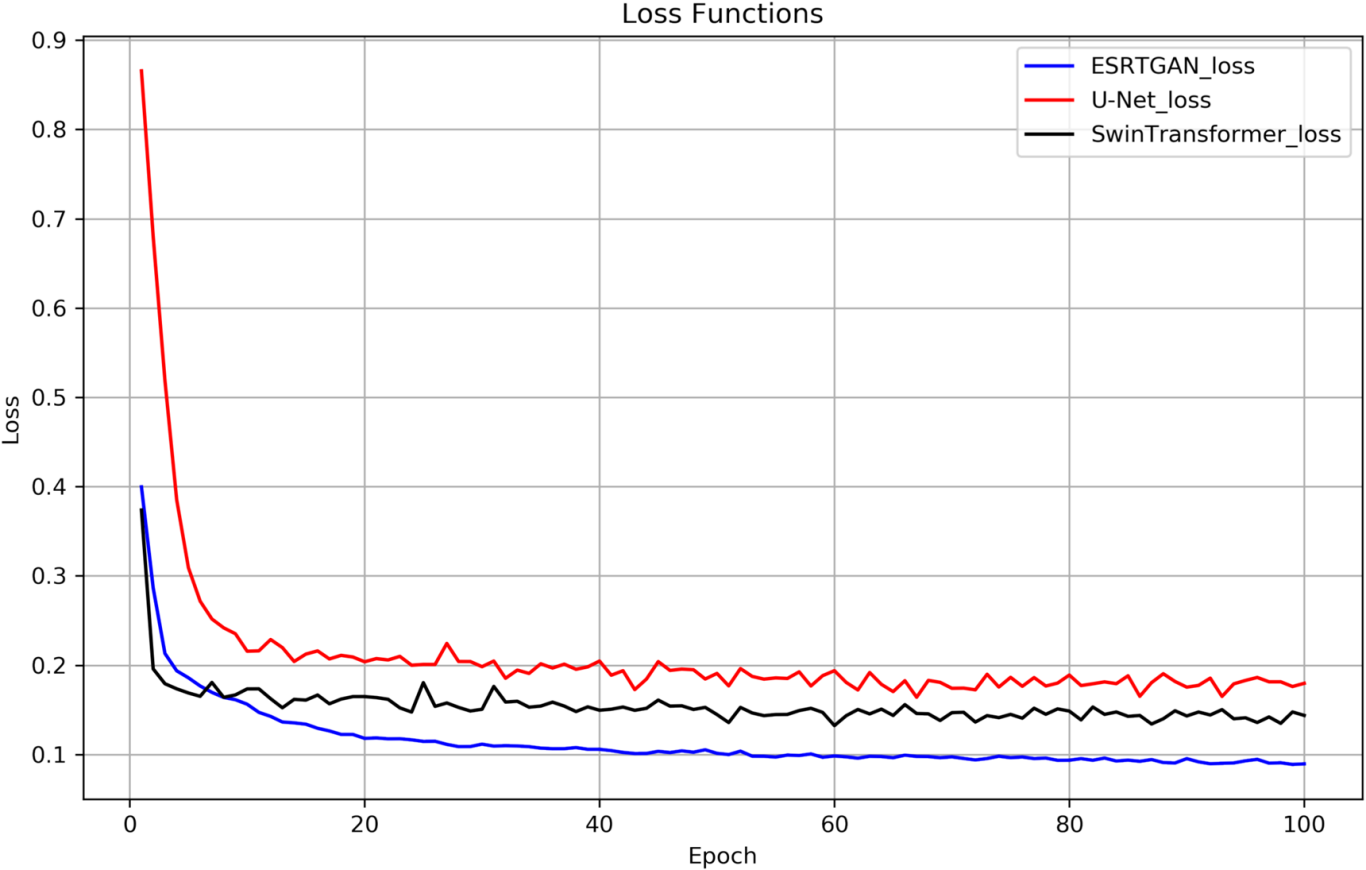
Training loss curves of U-Net, SwinTransformer, and the ESRTGAN.

## Experiments and results

### Data preparation

This study takes the Wujiang Dongfeng Hydropower Station, located at the border of Qingzhen and Qianxi counties in Guizhou Province, as the research object. This location is representative of underwater dam environments and is subject to common underwater imaging interferences such as turbidity and low light. The proposed ESRTGAN was trained using the PyTorch 1.13.0 framework on an NVIDIA RTX 4090 GPU. The dataset for training and evaluation was constructed by integrating public and self-collected image resources, while incorporating underwater optical transmission characteristics to ensure diversity and authenticity. Initially, 50 images were selected from the public underwater image dataset UFO120, covering basic underwater scene types. Additionally, 100 high-quality underwater images were captured by underwater robots at the Wujiang Dongfeng Hydropower Station (Guizhou, China), with training labels generated via bicubic upsampling and contrast/saturation adjustment to simulate high-resolution reference images. These 100 images all correspond to the concrete gravity dam of the Wujiang Dongfeng Hydropower Station, and fully cover the key underwater structural parts of the dam, including the upstream face of the dam body and the surface of the spillway. They can fully support the model in learning the degradation characteristics and enhancement rules of underwater images in different structural regions. The water depth during collection ranges from 5 to 20 meters, and the water turbidity remains stable.

To address the issue of insufficient sample diversity and enhance the model’s generalization ability, data augmentation operations were designed based on the physical mechanism of underwater image degradation: brightness was adjusted within the range of ±10% to ±15% to simulate light intensity changes in different underwater depths; blue and green channels were reasonably perturbed to mimic the color shift caused by water’s selective absorption of light; and horizontal flipping was applied to expand the dataset without altering structural features. Through these operations, the total number of images in the dataset was expanded to 3,000, which were used for model training. The trained model was evaluated on an independent test set consisting of additional images captured by underwater robots at the same hydropower station, ensuring the test data were not overlapping with the training set and could truly reflect the model’s practical application effect.

**Fig. 4:**
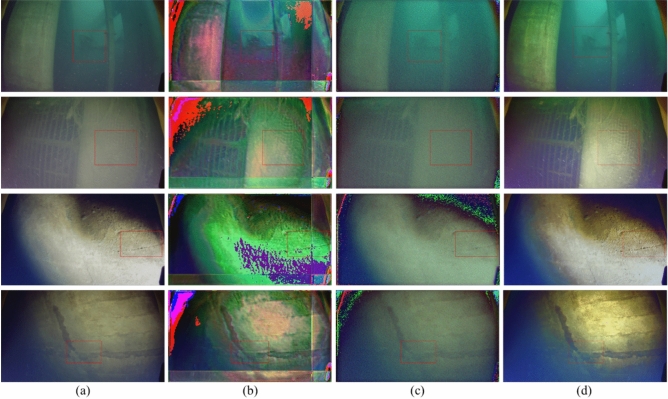
Effects of Image 1–4 processed by different methods; the red boxes indicate the zoom-in comparison regions, corresponding to the zoom-in comparison in Fig. 5. (**a**) Input; (**b**) U-Net; (**c**) SwinTransformer; (**d**) ESRTGAN.

**Fig. 5:**
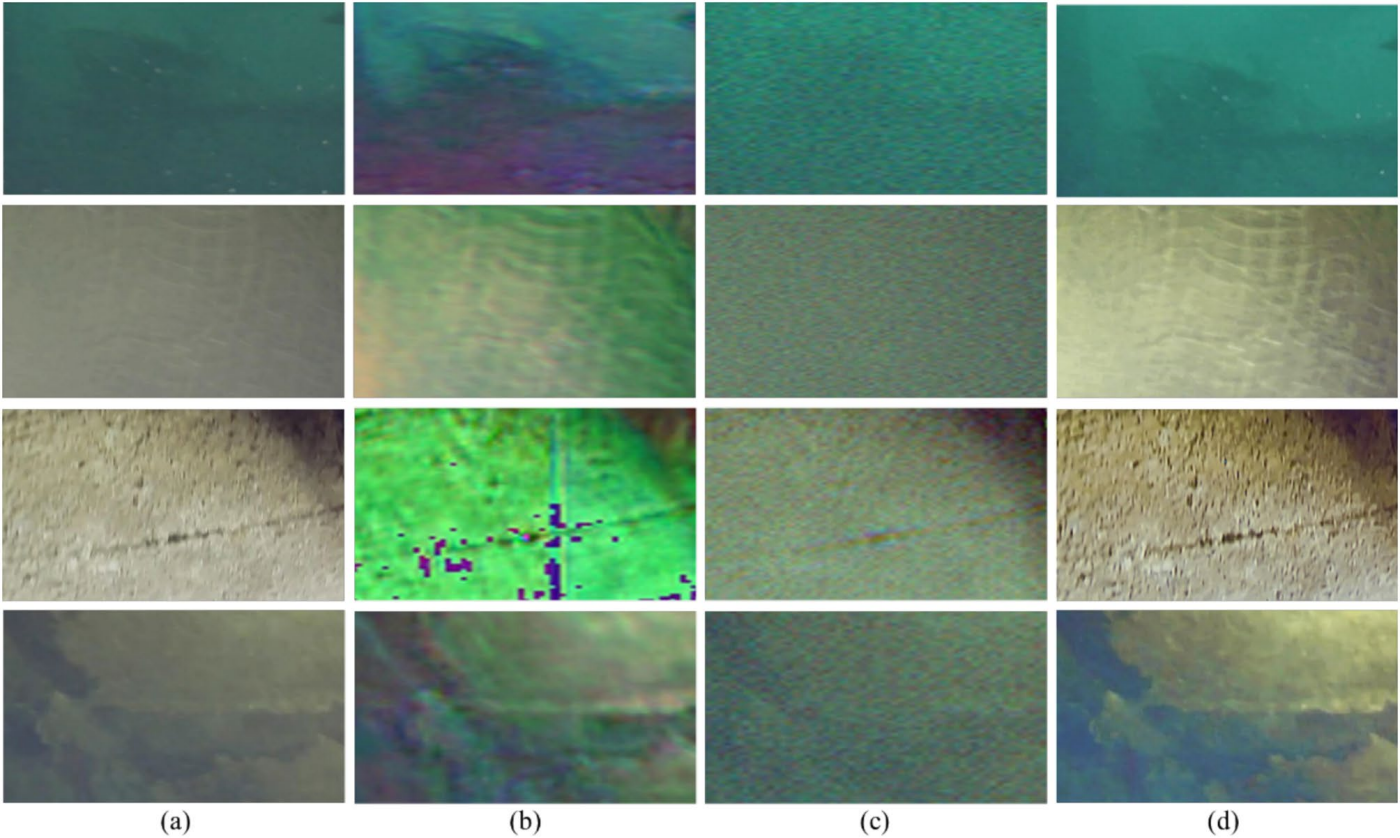
Zoomed-in comparison of red regions in Fig. 4. (**a**) Input; (**b**) U-Net; (**c**) SwinTransformer; (**d**) ESRTGAN.

All input images underwent unified preprocessing to standardize the input format for the network. First, raw images were cropped into 256$\times$256 pixel patches to match the input size requirement of the ESRTGAN generator and avoid excessive computational complexity caused by large-size images. Then, pixel value normalization was performed using a mean of 0.5 and a standard deviation of 0.5, scaling the pixel values from the original [0, 255] range to [−1, 1]. This normalization operation helps stabilize the network’s training process, reduce gradient fluctuations, and accelerate convergence. The super-resolution magnification factor was set to 2, meaning the generator outputs 512$\times$512 high-resolution images based on 256$\times$256 low-resolution input images, which meets the needs of image enhancement and improve the resolution.

The training hyperparameters were configured to balance model convergence speed, performance stability, and computational efficiency. The batch size was set to 1, considering the memory constraints of the training hardware and the need to ensure effective feature learning for each sample. The total training epoch was 100. For the optimizer, both the generator and discriminator adopted the AdamW optimizer to adaptively adjust learning rates and suppress overfitting. The generator learning rate was set to 2e-4, while the discriminator learning rate was 1e-4. The momentum parameters and of the AdamW optimizer were 0.9 and 0.999, respectively: controlled the exponential moving average of the first-order gradient moment to smooth gradient updates, and controlled the exponential moving average of the second-order gradient moment to dynamically adjust the learning rate adaptation range. A weight decay of 1e-4 was applied to both optimizers to regularize the model parameters and reduce overfitting to trivial background noise in underwater images.

The loss function for training integrated adversarial loss and content loss, with a weight coefficient was 0.05 to balance “visual realism” and “content fidelity”. The adversarial loss was calculated based on the Wasserstein distance to alleviate the training instability of traditional GANs, while the content loss was computed using the Euclidean distance between feature maps of generated and real images extracted by a pre-trained VGG network, ensuring the generated images retained the structural details of real dam underwater images.

### Method comparison

To verify the effectiveness of ESRTGAN, comparative experiments were conducted using U-Net^41^ and SwinTransformer^42^. Fig. 3 shows the training loss curves of U-Net, SwinTransformer, and the ESRTGAN generator. For fair comparison, ESRTGAN and the comparative methods were trained on the same dataset and tested on an independent test dataset. Performance results were provided to ensure the robustness of the model across different data distributions.

### Result comparison

Experiments were conducted on images captured by underwater robots at the Wujiang Dongfeng Hydropower Station (China). Full-reference image quality metrics—PSNR^43^, SSIM^44^, LPIPS^45^, UCIQE^46^, and UIQM^47^—were used to evaluate the experimental results of test images. Four underwater images were randomly selected from the image data collected by the underwater robot at the Dongfeng Hydropower Station on the Wujiang River, and all of these images are from the independent test set. The results of the experimental comparison are shown in Fig. 4, and the results show that compared with U-Net and SwinTransformer, ESRTGAN exhibits advantages in image denoising and preserving structural information and details of enhanced images. Column (a): Raw underwater images, which are dark overall, with obvious noise, blurred details, and monotonous colors, suffering from poor image quality due to the underwater environment. Column (b): Images processed by U-Net, showing improved color richness and relatively clearer edges/structural features, but with residual local noise and room for optimization in denoising. Column (c): Images processed by SwinTransformer, with significantly reduced noise, smoother frames, and enhanced overall structure/layering, but insufficient detail enhancement. Column (d): Images processed by ESRTGAN, featuring high color saturation, rich detail presentation, clear textures, and visual effects closer to the ideal clear state, achieving color restoration and detail enhancement.

**Table 1:** Quantitative comparison of different methods (PSNR$\uparrow$: higher values indicate better performance; SSIM$\uparrow$: higher values indicate better performance; LPIPS$\downarrow$: lower values indicate better performance).

Image	PSNR			SSIM			LPIPS		
	U-Net	SwinTransformer	ESRTGAN	U-Net	SwinTransformer	ESRTGAN	U-Net	SwinTransformer	ESRTGAN
Image 1	16.2246	23.3201	26.6429	0.8154	0.7616	0.9669	0.5266	0.4364	0.2692
Image 2	14.8186	19.6025	26.5325	0.7980	0.7507	0.9734	0.5148	0.4775	0.2818
Image 3	10.1549	13.0709	21.9434	0.6107	0.6188	0.9171	0.5827	0.5221	0.3102
Image 4	14.9216	18.6717	24.7448	0.7935	0.7237	0.9524	0.4782	0.4930	0.3122

**Table 2:** Quantitative comparison of different methods (UCIQE$\uparrow$: higher values indicate better performance; UIQM$\uparrow$: higher values indicate better performance).

	UCIQE				UIQM			
	Input	U-Net	SwinTransformer	ESRTGAN	Input	U-Net	SwinTransformer	ESRTGAN
Image 1	0.0646	0.0728	0.0969	0.1031	1.1795	1.5471	1.5443	1.5591
Image 2	0.0799	0.1129	0.1016	0.1301	1.1699	1.4171	1.3976	1.428
Image 3	0.1760	0.1839	0.1842	0.1869	1.5931	1.8051	1.8354	1.8563
Image 4	0.1013	0.1334	0.1299	0.1489	1.1924	1.5852	1.7158	1.7455

Quantitative evaluation (PSNR, SSIM, LPIPS) results are shown in Table 1. ESRTGAN achieved the highest PSNR/SSIM scores and the lowest LPIPS score, demonstrating advantages in fidelity. SwinTransformer outperformed U-Net in PSNR, while the two methods showed slight differences in SSIM and LPIPS. As can be seen from the Table 2, for different images, after being processed by the three methods, their UCIQE and UIQM values are all improved compared with those of the Input, but the improvements of UCIQE and UIQM values after being processed by ESRTGAN are the highest. In summary, ESRTGAN emphasizes the preservation of image structure and texture details, highlighting its effectiveness in enhancing structure-sensitive underwater images.

Fig. 5 shows the zoomed-in views of the red regions in Fig. 4. Images captured by underwater robots exhibit varying degrees of noise, blurriness, and difficulty in distinguishing texture details, with poor overall clarity: U-Net-processed images show obvious changes in color and contrast, with reduced noise in some regions but color distortion and blocking artifacts (e.g., purple/red patches in green regions), caused by overprocessing or misjudgment during model enhancement. SwinTransformer-processed images suppress noise to a certain extent and are relatively smooth but suffer from detail loss (e.g., cracks are less clear than in raw images), possibly due to over-smoothing during denoising. ESRTGAN-processed images show significant improvements in clarity and detail presentation, outperforming other methods in preserving original image features (e.g., cracks, textures) with more natural color and contrast.

Furthermore, under the same dataset conditions: U-Net and SwinTransformer (CNN-based) achieve limited denoising and enhancement effects. ESRTGAN (GAN-based) outperforms CNN-based methods in color restoration and detail enhancement, with visual effects closer to real scenes, demonstrating the superior performance of GANs in denoising and image enhancement for this underwater image processing task.

### Ablation experiments

To further verify the effectiveness of the proposed method, ablation experiments were conducted on ESRTGAN under different conditions. Under the same dataset training, the impact of different modules on underwater image enhancement was evaluated: ESRTGAN with the LightViT module removed, denoted as ESRGAN. On the basis of removing the LightViT module, remove the ChannelAttention in RRDB denoted as ESRGAN-A.

**Fig. 6:**
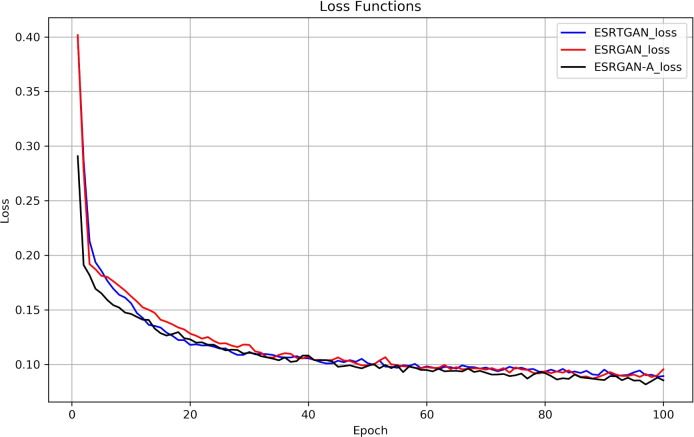
Comparison of loss function curves in ablation experiments.

Fig. 6 shows the loss curves of the three models. All models effectively reduced loss in the early training stage, indicating their ability to learn and optimize training data. In the late training stage: The ESRGAN-A loss curve showed small fluctuations but remained low. The ESRTGAN loss and ESRGAN loss curves also achieved good fitting.

**Fig. 7:**
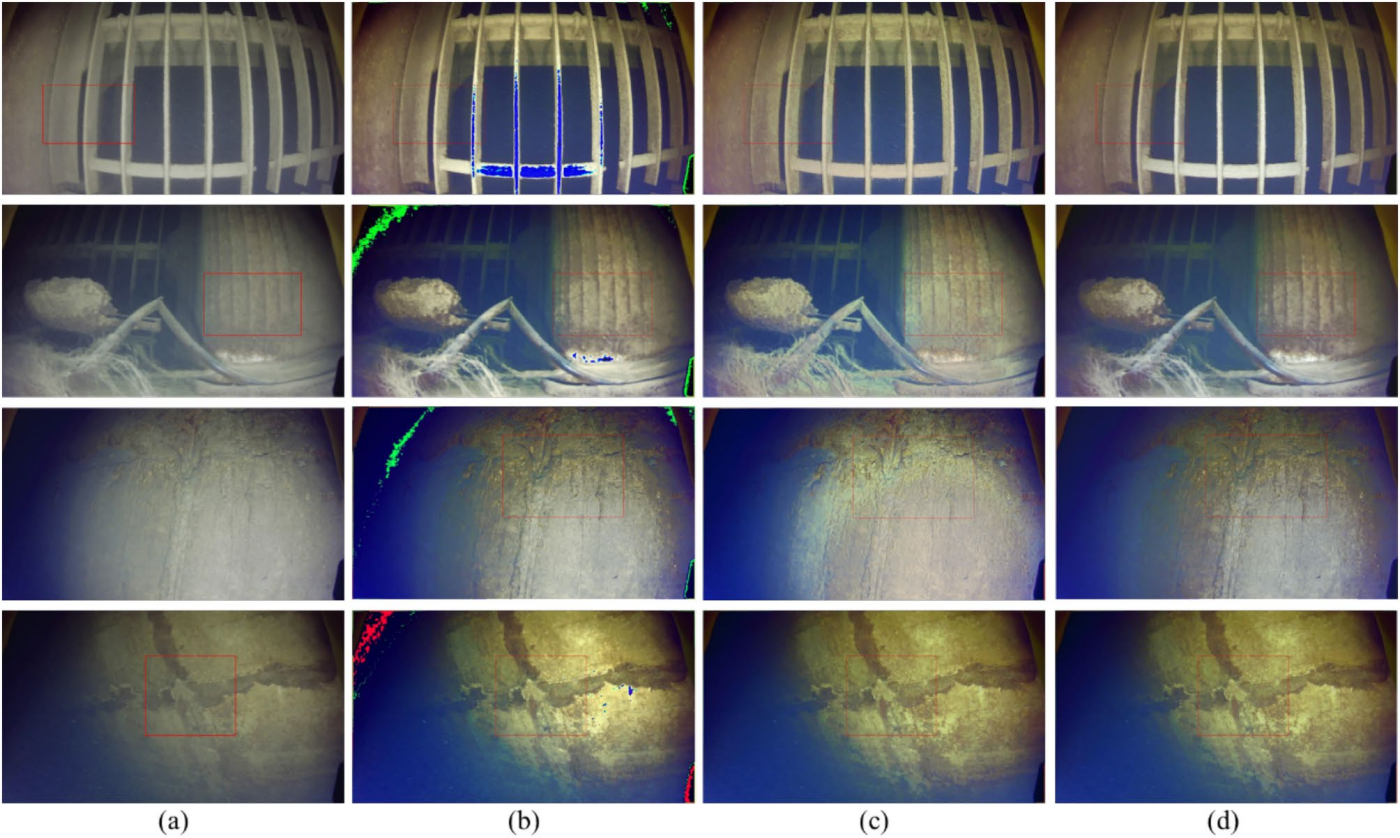
Effect of ablation experiment on images 1–4; the red box indicates the magnified comparison area corresponding to the magnified comparison in Fig. 8. (**a**) Input; (**b**) ESRGAN-A; (**c**) ESRGAN; (**d**) ESRTGAN.

The test data were still images captured by underwater robots at the Wujiang Dongfeng Hydropower Station. Fig. 7 provides a visual comparison of the three methods.

**Fig. 8:**
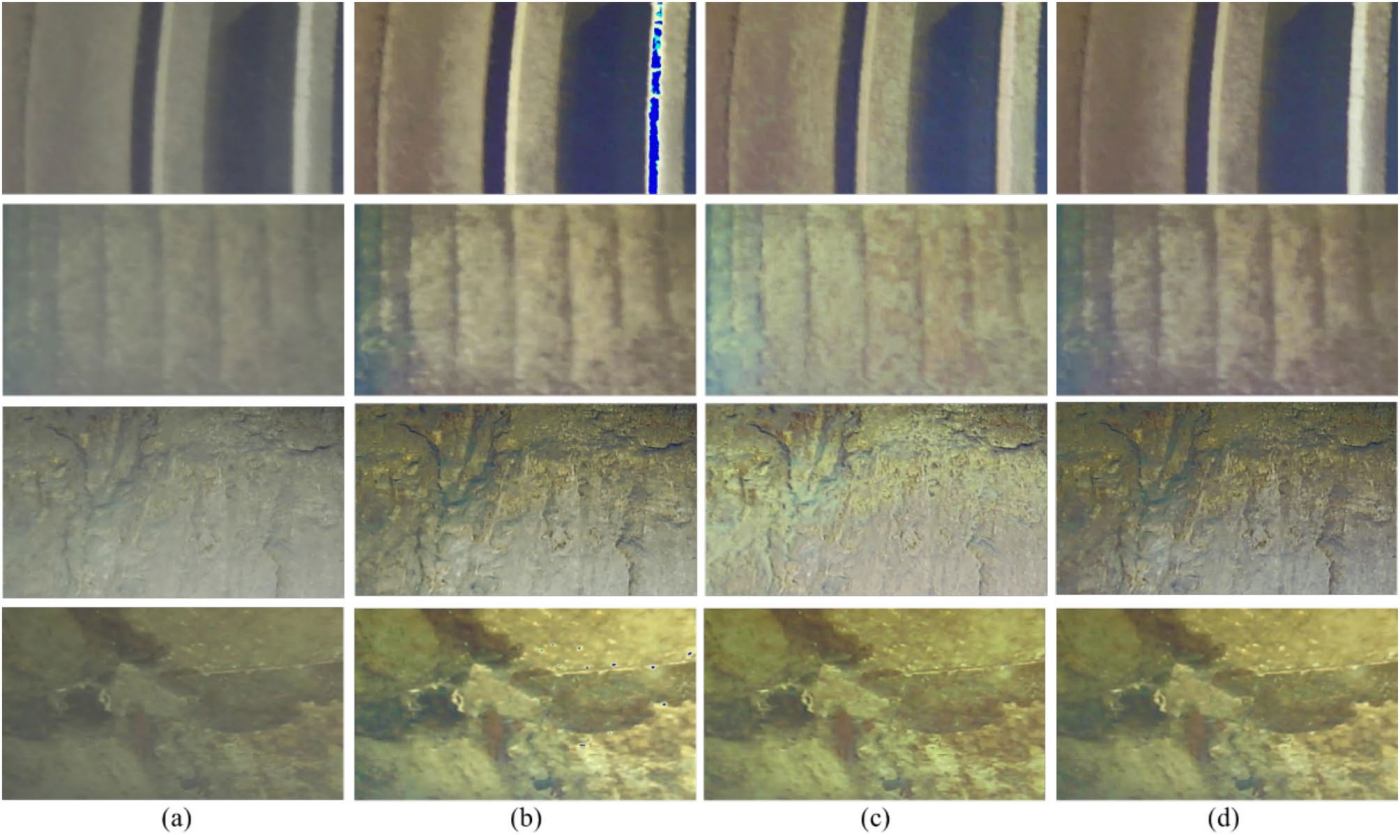
Zoomed-in comparison of red regions in Fig. 7. (**a**) Input; (**b**) ESRGAN-A; (**c**) ESRGAN; (**d**) ESRTGAN.

A visual examination of Fig. 7 reveals distinct performance differences among the four sets of images: Raw underwater images exhibit inherent limitations of underwater acquisition, including low overall clarity, poor contrast, prominent noise and blurriness, and nearly indistinguishable structural details; ESRGAN-A, while showing improved color richness and contrast relative to raw images—with enhanced presentation of fine features such as textures and cracks on dam structural surfaces—introduces unintended artifacts, specifically color distortion and blocking effects, which compromise both visual perception and the accuracy of structural information; ESRGAN effectively suppresses noise to produce smoother images but suffers from a critical trade-off: during the denoising process, it blurs key details, resulting in detail loss (e.g., cracks appear less distinct than in raw images and other processed results); in contrast, ESRTGAN demonstrates comprehensive superiority: it achieves superior overall clarity and detailed presentation, with clear visualization of texture and crack features on structural surfaces, maintains natural color tones and contrast without obvious color distortion or blocking artifacts, and balances effective denoising and enhancement with the preservation of original image structural features. Its visual effects are closer to real underwater scenes, thereby providing reliable image support for more accurate analysis of dam structural status and damage conditions.

**Table 3:** Quantitative comparison of ablation experiment results (PSNR$\uparrow$: higher values indicate better performance; SSIM$\uparrow$: higher values indicate better performance; LPIPS$\downarrow$: lower values indicate better performance).

Image	PSNR			SSIM			LPIPS		
	ESRGAN-A	ESRGAN	ESRTGAN	ESRGAN-A	ESRGAN	ESRTGAN	ESRGAN-A	ESRGAN	ESRTGAN
Image 1	23.8934	25.8407	26.5934	0.9093	0.9690	0.9708	0.3204	0.2885	0.2562
Image 2	24.3575	26.5095	26.7149	0.9156	0.9534	0.9653	0.3364	0.3257	0.2948
Image 3	25.9954	26.7552	26.8688	0.9334	0.9574	0.9598	0.3236	0.2934	0.2922
Image 4	25.1108	26.5479	26.6858	0.9227	0.9703	0.9733	0.3225	0.3125	0.2698

**Table 4:** Quantitative comparison of ablation experiment results (UCIQE$\uparrow$: higher values indicate better performance; UIQM$\uparrow$: higher values indicate better performance).

	UCIQE				UIQM			
	Input	ESRGAN-A	ESRGAN	ESRTGAN	Input	ESRGAN-A	ESRGAN	ESRTGAN
Image 1	0.0933	0.1271	0.1126	0.1207	1.2867	1.5545	1.5464	1.5743
Image 2	0.1106	0.1524	0.1247	0.1456	1.2342	1.4965	1.4763	1.5602
Image 3	0.1486	0.1747	0.1437	0.1715	1.4528	1.6562	1.5054	1.8135
Image 4	0.0936	0.1185	0.1299	0.1769	1.1493	1.5964	1.5909	1.6908

Quantitative indicators (PSNR, SSIM, LPIPS) for ablation experiments are shown in Table 3. ESRTGAN exhibited outstanding performance in PSNR and LPIPS, achieving good results in reducing image distortion and maintaining visual perception similarity. ESRGAN also performed well in SSIM, effectively preserving image structural similarity. It can be seen from Table 4 that the performance of different methods (ESRGAN-A, ESRGAN, and ESRTGAN) is evaluated using two metrics, UCIQE and UIQM. For both metrics, the higher the value, the better the performance. Looking at the UCIQE column and the UIQM column, for Image 1 to Image 4, the UCIQE values of the images processed by ESRGAN-A, ESRGAN, and ESRTGAN are all higher than those of the input images. This indicates that, from the perspective of the UCIQE metric, all three methods can improve the quality of the input images. Moreover, among these three methods, ESRTGAN continuously achieves the highest UCIQE and UIQM values for each image.

Fig. 8 presents the zoomed-in views of the red regions in Fig. 7, from which the differences in image enhancement effects among various algorithms can be clearly observed. The ESRGAN-A algorithm enhanced certain details such as textures and cracks, yet it simultaneously caused color distortion. The ESRGAN algorithm performed well in improving image clarity and reducing noise, while also maintaining natural color tones. However, it fell short in terms of detail sharpness—for instance, the edges of fine cracks appeared blurry. In contrast, the ESRTGAN algorithm achieved a further improvement in clarity: it not only delivered rich and natural details, making texture and crack features clearly distinguishable, but also exhibited high color restoration accuracy. There were no obvious distortion phenomena or abnormal color blocks, and it well preserved the realism of the image while enhancing its details.

In conclusion, ESRTGAN exhibits a significant effect on underwater image denoising and enhancement. Ablation experiments verify that removing key modules leads to issues such as color distortion and detail loss, confirming the importance of each module in ESRTGAN for improving color fidelity and detail clarity of underwater images.

## Conclusion

This study proposes the ESRTGAN method to address the problem of denoising and enhancement of dam underwater images, aiming to solve the challenge that optical degradation of underwater images affects the accuracy of dam safety assessments.

The proposed method fuses the local feature extraction of CNN and the global context modeling of Vision Transformer to construct the ESRTGAN network. The generator includes shallow feature, deep feature, and image reconstruction modules, improving detail reconstruction capability via multi-scale feature fusion, adaptive channel attention, and progressive training. The discriminator effectively captures multi-scale features using convolutional downsampling and BatchNorm, providing supervision signals for the generator. The loss function combines adversarial loss and content loss to guide the generator’s optimization.

Under the same experimental conditions, compared with U-Net and SwinTransformer, ESRTGAN achieves higher PSNR/SSIM scores and lower LPIPS scores, with superior subjective visual effects, demonstrating significant advantages in denoising, color restoration, and detail enhancement. Ablation experiments show that removing key modules causes color distortion and detail loss, verifying the importance of each module in improving color fidelity and detail clarity of underwater images.

However, like any cutting-edge research, although this work has achieved phased breakthroughs, there is still room for improvement. It is necessary to objectively analyze the current limitations to present a more balanced research perspective: First, the study has a certain dependence on synthetic data. The dataset of this study mainly consists of two parts: one is 100 real underwater images collected from the Wujiang Dongfeng Hydropower Station, and the other is 50 images from the public dataset UFO120. To address the insufficient sample diversity, the study expanded the sample size to 3000 images through data augmentation operations. Although these synthetic samples are designed based on the physical mechanism of underwater image degradation, they still cannot fully simulate the complexity and variability of the real underwater environment, which may lead to a certain gap between the model’s enhancement effects on synthetic samples and those in real extreme scenarios.

Second, in terms of computational cost, the model is not yet suitable for real-time image enhancement needs. ESRTGAN adopts a fused architecture of RRDB and LightViT. Although this study reduces computational complexity through fixed 7$\times$7 window attention, the model still has high requirements for hardware resources.

Third, the model’s adaptability to different water qualities needs to be improved. Since the study is applied to the specific Wujiang Dongfeng Hydropower Station, our dataset and parameter settings are all designed for the underwater environment of this hydropower station. Therefore, the parameters of the experimental dataset are relatively single. In extreme water quality conditions, the model may have problems such as insufficient noise suppression or excessive color correction.

Future research can delve into the following key directions: First, regarding model lightweighting research, the current model’s operational efficiency needs to be improved in practical applications, especially on embedded devices such as underwater robots that have strict requirements for computing resources and processing speed. In the future, we can try to replace some standard convolutions in the model with depthwise separable convolutions to significantly reduce the number of parameters. At the same time, we will optimize and prune the model structure to remove redundant parts and reduce computational complexity. In addition, knowledge distillation technology will be introduced to significantly improve the model’s running speed while ensuring the basic stability of the image enhancement effect, making it better adapt to the real-time processing needs of embedded devices.

Second, for multimodal fusion exploration, the underwater environment is complex and changeable, and a single optical image is often difficult to fully reflect the actual situation. In the future, we can consider fusing multiple data sources. For example, data such as turbidity and pH value fed back in real time by water quality sensors can also be used to build a mapping relationship between environmental parameters and image enhancement strategies, so that the model can intelligently adjust enhancement parameters according to different water quality conditions, further improving the adaptability and accuracy of the enhancement effect.

Finally, for dataset expansion, the currently used dataset has certain limitations in scene richness and sample diversity. In the future, we plan to unite multiple parties to extensively collect underwater images covering various scenarios such as different water area types, light conditions, turbidity levels, and biological distributions, and build a large-scale and comprehensive underwater image enhancement benchmark dataset. At the same time, domain adaptive training technology will be used to train and optimize the model on datasets from different sources and with different characteristics, reducing the model’s overfitting to specific scenarios and improving its universality and reliability in various complex underwater scenarios, thereby laying a more solid data foundation for the practical application of underwater image enhancement technology.

To sum up, the underwater image enhancement algorithm proposed in this study has achieved certain results and provided new ideas and methods for the development of this field. The subsequent clear research directions will continue to promote the innovation of underwater image enhancement technology, making it better serve many practical application fields such as underwater engineering and ecological protection.

## Data Availability

The dataset used in this study can be obtained from the author yuanchongxin and reasonable requests can be made by email ycx@cwnu.edu.cn.
